# Effects of a 12-Week Mixed-Method Physical Exercise Program on Physical Fitness, Stress, Anxiety, and Quality of Life in Adolescents with Cerebral Palsy: A Case Series Study

**DOI:** 10.3390/children11101257

**Published:** 2024-10-18

**Authors:** Alexandrina Cavalcante Rodrigues Nitz, José Pedro Ferreira, Elaine Maria Ribeiro, Juliana Albuquerque da Rocha, Chrystiane Vasconcelos Andrade Toscano, Maria João Campos

**Affiliations:** 1 Faculty of Sport Sciences and Physical Education, University of Coimbra, 3040-248 Coimbra, Portugal; 500883@sarah.br; 2 Sarah Network of Hospitals of Rehabilitation, Fortaleza 60861-634, Brazil; 13283@sarah.br (E.M.R.); julianarocha@sarah.br (J.A.d.R.); 3 Research Unit for Sport and Physical Activity, Faculty of Sport Sciences and Physical Education, University of Coimbra, 3040-248 Coimbra, Portugal; jpferreira@fcdef.uc.pt; 4 Physical Exercise Research Project for People with Autism Spectrum Disorder (PEFaut), Institute of Physical Education and Sport, Federal University of Alagoas (UFAL), Maceio 57072-970, Brazil; chrystiane.toscano@iefe.ufal.br

**Keywords:** physical exercise program, well-being, neurodevelopmental disorders, teenagers, rehabilitation

## Abstract

Background/Objectives: Although the health benefits related to physical exercise for adolescents with cerebral palsy (CP) have been recognized, studies indicate that individuals with CP at school age are less involved in physical activities than their typical peers and are twice as likely to engage in sedentary behaviors. Therefore, our study aims to investigate the effects of a physical exercise program on physical fitness, stress, anxiety, and quality-of-life variables. Methods: A total of 15 teenagers with ambulatory CP (*n* = 8 boys, *n* = 7 girls, between 12 and 18 years old; M = 14.35; SD = 1.76) completed a 12-week program based on a mixed-method approach with face-to-face and live online activities. The outcome measures were physical fitness, stress, anxiety, and quality of life. Results: The 12-week exercise program resulted in gains in muscular strength, flexibility, and aerobic endurance tests, characterized by an increase in average walking speed and average VO_2_ max. There was also a significant change in the perception of emotional states of depression, anxiety, and stress reported by the participants. Conclusions: The program proved to be effective in physical fitness tests and perception of emotional states. Given the positive effects produced by the program, its design appears to meet the demands of adolescents with cerebral palsy.

## 1. Introduction

Cerebral palsy (CP) is one of the most common neurodevelopmental disorders caused by non-progressive damage to the immature brain [1]. Its estimated incidence is 1.4 to 1.8 in 1000 live births in industrialized countries [2]. The degree of motor impairment varies from those who have an almost normal function to others who have a significant disability. The clinical characteristics and comorbidities of CP are also varied. About 75% of children experience pain, 50% have an intellectual disability, at least 25% have epilepsy, and about 20% to 25% have behavioral or sleep disorders [3]. In addition to clinical comorbidities, the occurrence of stress and anxiety in adolescents with CP has been identified [4]. Impacts on quality of life were identified in the physical, psychological, and social domains [5].

Although CP is a non-progressive condition, it results in primary and secondary disabilities, such as muscle changes, decreased range of joint movement, impaired balance function, and reduced cardiorespiratory endurance [1,6]. Consequently, children and adolescents may also present worse performance in daily activities and restricted participation [7].

One of the main therapeutic goals for children and adolescents with CP is improving motor function [8]. Over the past decade, the benefits of physical activity (PA) have become progressively clear [9]. Research shows that being physically active benefits everyone, regardless of personal and environmental factors [10,11]. It has been reported that a minimal increase in PA could improve overall health [12]. The practice of PA is also related to better mood, better mental health, well-being, and less stress [13,14]. Historically, the recommendations and benefits of physical activity/exercise for individuals with CP have not been clearly defined [15]. However, research suggests that individuals with CP who participate in sports or exercise experience the health benefits reported in the general population [15]. The effects may depend on the proposed activity and the training parameters to achieve the objectives. Specific interventions are aimed at improving muscular strength [16], balance [17], and aerobic capacity [18]. The relationship between physical exercise and the quality of life of children and adolescents with CP has also been investigated [19]. Unfortunately, evidence suggests that school-aged children with CP are 30% less involved in PA than their typical peers and twice as likely to engage in sedentary behavior [20]. Adherence to exercise programs is challenging, especially in children and young people with more significant motor impairments, which limit independent mobility. Success in adhering to the activity depends on the commitment and availability of both the patient and the caregiver [15]. Many barriers that make physical exercise difficult justify a sedentary lifestyle [21]. In this population, intellectual impairment, the presence of pain during exercise, and the daily challenges of managing a child with disability are some reported barriers to participating in PA [22,23].

Home exercises began to be studied to improve exercise adherence. Exercising at home was considered a facilitating strategy for those who had difficulty accessing sports halls or gyms or who were embarrassed to exercise in public [24]. Due to protective measures and restrictions, home exercise has been encouraged, especially during the COVID-19 pandemic [25]. In these extreme circumstances, the disruption of access to healthcare and community support services has also affected people with disabilities and their families, resulting in increased stress and anxiety and decreased PA [26,27]. Technological advances have opened up new possibilities for home exercise programs. Online exercise prescription websites and apps have seen significant improvements. Telemedicine, in particular, has made great strides in the face of the COVID-19 pandemic. It is now possible to conduct home exercises online, with the live supervision of a specialized professional, offering a promising alternative to traditional in-person programs. Following the COVID-19 pandemic, these programs may be synchronous (e.g., video conferencing with a professional), asynchronous (e.g., exercises with digital images and videos), or hybrid (e.g., a combination of synchronous and asynchronous). Studies describe the positive effects of home exercises for older adults and consider them a viable and acceptable alternative to face-to-face programs [28]. They showed that Internet-based programs can offer an alternative for participants who cannot attend regular face-to-face physical activity programs. However, another study raises the limitations of home-based PA in terms of social participation, recreation, and leisure programs for children with CP [25]. Another study suggests that online or hybrid exercise programs incorporating interactive methods (i.e., competition and cooperation) can improve social interaction and quality of life among school-aged children with CP who use wheelchairs [29].

Given the recent changes in exercise programs and the lack of comprehensive answers in the literature, we aim to investigate the potential effects of a 12-week intervention based on physical exercise (PE) in a mixed-method program that alternates face-to-face and live online activities on physical fitness, stress, anxiety, and quality of life of adolescents with cerebral palsy.

## 2. Materials and Methods

### 2.1. Participants

Fifteen participants with ambulatory CP (*n* = 15), eight boys and seven girls, between 12 and 18 years old (M = 14.35; SD = 1.76), met the criteria to participate in the final analysis. Most adolescents had CP GMFCS level I (60%, *n* = 9) and the most common topography was diplegia (53%, *n* = 8).

#### 2.1.1. Inclusion Criteria

Eligibility criteria for participation included the following:

(i) A medical diagnosis of CP (information collected in electronic medical records).
(ii) An age between 12 and 18 years.
(iii) CP classified as level I, II, or III by the Gross Motor Function Classification System (GMFCS) and an ability to walk, even if the assistance of a device is required [30].
(iv) The ability to be monitored in a rehabilitation center.
(v) Live in Fortaleza, Brazil, or nearby cities to enable face-to-face monitoring.

#### 2.1.2. Exclusion Criteria

Adolescents with selected age and diagnosis who met the following criteria were excluded from the study:

(i) Those who did not agree to participate in the PE program or whose parents or legal representatives did not authorize their participation in this study.
(ii) Those who had clinical contraindications for participation in the proposed PE program or were using any corticosteroid medication that could confuse the study results (information in electronic medical records).
(iii) Those who had undergone neurological surgery or invasive/non-invasive orthopedic intervention in the last six months (information in electronic medical records).
(iv) Federated athletes.
(v) Those who were unable to provide sufficient cooperation in intervention and testing sessions.

The program was 50% face-to-face and 50% online, and to evaluate the intended effects, we established a 75% attendance rate for the activities. The data evaluated corresponded to data from 60% of the sample since 40% of the adolescents missed more than 25% of the program. The absence was usually in face-to-face activity due to financial difficulties in traveling to the rehabilitation center or the illness of the primary caregiver. Two adolescents dropped out of the program due to a lack of interest in physical activity.

### 2.2. Ethic

This research was conducted following the recommendations of the ethical guidelines for research with human beings and the Resolution of the National Health Council. An information session was held with the adolescents and their parents or legal representatives to explain the study’s objective, data collection, and the characteristics of the intervention program; those who agreed to participate provided written informed consent according to the Declaration of Helsinki. The research was approved by the Rede Sarah Ethics Committee on 6 April 2023 (CAAE 63680022.5.0000.0022).

### 2.3. Study Design and Procedures

A case series study was conducted in a rehabilitation center (Sarah Network of Rehabilitation Hospitals—Sarah Fortaleza) between August and November 2023.

Participants were invited to face-to-face sessions once a week in the rehabilitation center and online, also once a week, during regular opening hours. Training sessions lasted 45 min and were separated by at least 48 h. The intervention program lasted 12 weeks (24 sessions). Adolescents were grouped by motor ability. Each group had up to 6 adolescents and two mediators, from the research team.

Attendance was recorded and, in the case of two consecutive absences, an institutional employee called or sent a message to the legal guardian to identify the reasons related to the absences. Participants who attended at least 75% of the 24 sessions (at least 9 face-to-face sessions and at least 9 online sessions) were included in the study and considered for data analysis.

The exercise program was based on the program described by Ferreira et al. (2018) [31] in their study protocol with children and young people with autism spectrum disorder (ASD), which aimed to improve physical fitness in the dimensions of aerobic endurance and muscular strength [31]. A third dimension was added to this study—flexibility.

Regarding the intensity of the exercises, the patients were monitored by heart rate, based on the initial assessment. The exercises ranged from moderate to intense intensity (maximum 75% of HR). During face-to-face training, patients wore a smartwatch with GPS to monitor their heart rate. Adolescents and caregivers were instructed to measure heart rate at home using the stopwatch on their own watch or mobile phone. The exercises were moderate to intense.

The muscle-strengthening training with elastic bands was progressive, with each teenager improving in resistance and, therefore, changing the elastic band individually. The muscular resistance training, with one’s own body weight, was progressive in terms of the number of repetitions, also on an individual basis, respecting the possibilities of each teenager.

Each training session began with a preparatory phase (5 min) in which the adolescent was prepared for the exercise session, including a brief conversation and warm-up.

In the development phase (30 min), the teenager performed cardiorespiratory endurance, muscular endurance, and strength exercises. At this stage, the exercises were diversified and organized in a progressive sequence of difficulty to maintain the teenagers’ interest and fun. Motivational strategies included different materials, different locations within the rehabilitation hospital, and music. Such strategies have been used to help encourage adherence and reduce barriers.

In the return to the calm phase (10 min), the teenager performed stretching and relaxation exercises.

A physiotherapist with a complete understanding of the training protocol supervised all sessions. Considering the possibility of cognitive impairment, participants were taught how to perform every exercise face-to-face and online.

To carry out the activities at home, the adolescents needed a cell phone (with a camera), Internet access, a mattress, a ball, and a regular chair. The online groups comprised six teenagers, supervised by a physiotherapist and a physiotherapy intern. If necessary, a family member was requested to be present. The sessions were held in a live-streaming format, so both staff and patients could see each other and staff could monitor patient exercise performance. An overview of the physical exercise program is given in Table 1. Due to the large volume of exercises, it was not possible to describe how each exercise was performed. The exercises were alternated. For example, in some sessions, there were yoga classes, and in others, there was stretching.

### 2.4. Data Collection Procedures

A multidisciplinary team that included physiotherapists, a pediatrician, and a biomedical practitioner with professional experience in CP services formed the intervention team.

The evaluations were meticulously planned and executed by the main investigator and the research team members. To ensure consistency, the same evaluators collected data at both assessment moments. Adolescents evaluated by a specific physiotherapist underwent the intervention program with another physiotherapist to further minimize the risk of bias.

An accredited biomedical practitioner performed the salivary collection four times: shortly before (8 a.m.) and shortly after (9 a.m.) the first PE session, and shortly before (8 a.m.) and shortly after (9 a.m.) the last PE session.

The remaining measures were collected twice, one week before the first PE session and one week after the last PE session.

### 2.5. Outcome Measures

The effect of the exercise program on physical fitness, stress, anxiety and quality of life of adolescents with CP was assessed using pre-test and post-test measures, which were crucial in determining the program’s impact.

The primary outcomes are related to physical fitness and body structure and function. Secondary outcomes are related to stress and anxiety levels and the perception of health-related quality of life.

#### 2.5.1. Anthropometric Measurements

All anthropometric/body composition measurements were taken by a single experienced observer following standardized procedures [32].

Anthropometric measurements were performed following standardized procedures. Measurements were taken individually. A portable stadiometer model P200C-LD105 Lider (Araçatuba, São Paulo, Brazil), measured body mass and height. The participant stood barefoot on the stadiometer platform, looking straight ahead with their arms at their sides. The body mass index from the formula weight (kg)/height (m^2^) was then calculated. Waist circumference was also measured halfway between the iliac crest and the tenth rib, directly on the skin, using a flexible measuring tape. The methods are viable, reliable, and accurate for the population.

#### 2.5.2. Functional Capacity

Physical fitness assessment was based almost entirely on applying a set of tests selected from the Brockport Battery Physical Fitness Test—BBPFT [33]. The tests were chosen according to the need to respond to the proposed intervention and the possibility of reliable performance by the participants. The BBPFT is validated for the study.

According to the objective, we assess physical fitness in cardiorespiratory endurance, muscular endurance/strength, and flexibility. The following tests were selected:

##### Modified Curl-Up [33]

The objective of this test is to estimate the degree of abdominal strength.

Repetitions are performed with a cadence of 1 to 3 s (cadence can be provided by clapping). The subject performs the repetitions until exhaustion or up to 75.

##### Trunk Lift [33]

The test aims to measure the trunk and neck extension capacity.

This test evaluates the distance from the floor to the chin. Two attempts are allowed, and the best score is recorded. The maximum score is 12 inches (30.48 cm).

##### Back-Saver Sit and Reach Test [33]

The test measures the flexibility of the lower back and hamstring muscles. The score is the farthest point (in centimeters) reached with your fingertips. The score corresponding to the best of three attempts is recorded.

##### Shoulder Stretch Test [33]

The aim is to test the flexibility of the shoulder joint.

If the fingers of both hands touch, “Yes” (S) should be recorded. If the subject cannot touch their fingers, the result to be recorded will be “No” (N), and the minimum distance between the hands will be measured. Repeat, changing the position of your arms.

##### Aerobic Fitness Test—6 min Walk Test (6MWT) [33]

This test assesses aerobic capacity and endurance. The “6 min walk test” is valid and reliable for evaluating the aerobic resistance of the studied population. Participants can stop and rest, then sit down and resume the route if necessary. Average speed and heartbeats per minute were measured (before and after the test) using a smartwatch.

##### Aerobic Endurance Test—Step Test [34]

This submaximal test aims to provide a measure of cardiorespiratory or endurance fitness. The Queens College Step Test was added to the test battery as an alternative to the target aerobic movement test (TAMT–BBPFT), as it is shorter and, therefore, more suitable for the selected population. The test was performed in a fixed surface, and light support was allowed for adolescents due to the risk/fear of falling. The subject moves up and down the platform using a four-step, ‘up–up–down–down’ cadence for 3 min. Heartbeats are counted for one minute (bpm) before and immediately after the end of the test. An estimate of VO_2_ max is calculated from the test results using a specific formula.

#### 2.5.3. Depression, Anxiety, and Stress Scale

The 21-item short version of the Depression, Anxiety, and Stress Scale (DASS-21) was developed by Lovibond and Lovibond [35]. It is a set of three scales designed to measure and differentiate the emotional states of depression, anxiety, and stress. This shortened version of the DASS was developed to reduce administration time and can be used for clinical investigation and research purposes. It is a self-report scale. The literature presents strong evidence that the scales are stable over time [36] and respond to treatment for mood problems. The scale was translated and validated in the context of Brazilian adolescents [37].

Each of the three DASS-21 scales contains seven items. When filling out the DASS-21, the interviewee is encouraged to check for the presence of any symptoms in the previous week. Each item is scored from 0 (does not apply to me) to 4 (applies very much or most of the time). The result for depression, anxiety, and stress is calculated by summing the item scores. According to the score, the symptom classification can be standard, mild, moderate, severe, or extremely severe.

#### 2.5.4. Biological Response of Physical Activity to Stress and Anxiety

Physiological stress markers, such as salivary cortisol levels and heart rate variability, objectively measure changes in the stress response. Salivary cortisol is a valid and reliable reflection of the respective unbound hormones in the blood. It reflects changes in serum cortisol levels, with the advantage of being a minimally invasive procedure [38].

Salivary cortisol measurements were collected as a physiological marker of participants’ stress levels. These data were collected on a different day from the other assessments due to the need to collect such data immediately before and after physical exercise. Saliva samples were obtained using the Salivette Cortisol device, Euroimmun (Lübeck, Germany). Participants placed the cotton removed from the Salivette tube in their mouths. The sample absorption time was 2 min. Once recovered, the device was centrifuged at 1000× *g* for 2 min. After centrifugation, the sample obtained was placed in an Eppendorf-type plastic tube for refrigeration (between 2 and 8 degrees Celsius) for 14 days after collection until testing. The measurements were carried out in duplicate implementing the ELISA methodology using the Cortisol in Saliva reagent kit from the manufacturer Euroimmun (Lübeck, Germany). The automatic microplate washer model ION-B-BI, Wuxi Hiwell Diatek Instruments Co., Ltd. (Wuxi, China) was used, and reading was carried out on a microplate reader model TP Reader NM, Thermoplate (Troy, MI, USA). The residual material was treated as standard biological waste.

#### 2.5.5. Health-Related Quality of Life—KIDSCREEN-10

KIDSCREEN is a fully open access questionnaire developed for clinical studies and research projects [39]. The 10-item version, KIDSCREEN-10, is the shortest of the three KIDSCREEN questionnaires and measures overall health-related quality of life (HRQoL). It can be answered in a few minutes by healthy adolescents or those with a diagnosis (self-assessment). A corresponding version is available for parents (proxy trial).

The KIDSCREEN-10 index covers physical well-being, emotions, autonomy, family/friends, and school environment, with two items for each segment. It is recommended that the teenager and family/guardian mention the last week in their answers. The instrument provides a single HRQoL score, with higher scores representing better HRQoL. It has good discriminatory power along the HRQoL trait continuum. Its psychometric properties in adolescents aged 12 to 18 are considered adequate [39]. The questionnaire was translated into Brazilian Portuguese and validated for the Brazilian population [40].

#### 2.5.6. Complementary Questions

A complementary questionnaire was evaluated, following the guidelines of the International Classification of Functioning, Disability and Health (ICF) [41]. It was used to characterize the adolescent’s personal factors, use of orthosis, routine, means of transportation to the rehabilitation center, and social situation. A parent or caregiver answered supplementary questions.

### 2.6. Data Analysis

Descriptive statistics will be presented on the participants’ personal and environmental data, such as age, sex, education, use of orthosis, participation in physical activity, recreational activity, and transportation conditions (public/private). Such data were collected through a semi-structured interview research format.

Normally distributed continuous variables are presented as the mean and standard deviation. Variables with non-normal distribution are presented as medians and interquartile ranges. The normality of the outcome variables was tested using the Shapiro–Wilk test. Comparisons between groups were performed using the *t*-test, Pearson’s chi-square test, or the Kolmogorov–Smirnov test. A significance level lower than 0.05 was considered statistically significant. The statistical program SPSS version 21 was used for the analyses.

## 3. Results

Fifteen teenagers with ambulatory CP (14.35 ± 1.76 years old), 8 boys and 7 girls, successfully completed the mixed 12-week exercise intervention program. No adverse events were identified during the intervention. Table 2 presents the baseline characteristics of study participants, including PA, recreational, and mobility habits.

Table 3 presents the mean and standard deviation values, and mean differences pre- and post-intervention with exercise, of the body composition, physical fitness, salivary cortisol levels, and quality of life of participants with CP, calculated with a *t*-test.

### 3.1. Body Composition

Regarding body mass, weight, height, BMI, and WC variables, no significant statistical differences were found pre- and post-intervention with exercise; however, for WC, there was a small Δ_mean_ variation with a positive overall reduction of about 0.5 cm.

### 3.2. Physical Fitness Tests

In terms of physical fitness variables, significant statistical differences were found pre- and post-intervention with exercise.

#### 3.2.1. Modified Curl-Up

The average number of repetitions increased from 22.73 to 62.12, respectively, showing a statistically significant increase in abdominal strength (*p* < 0.001). Cohen’s d = 2.13 (large effect size, indicating a significant improvement in the number of curl repetitions after training), suggesting a relevant impact of training on muscular strength and endurance.

#### 3.2.2. Trunk Lift

The average value of trunk lift increased from 22.73 to 28.53 cm, respectively, showing a statistically significant increase in upper body strength (*p* < 0.01). Cohen’s d = 1.06 (large effect size, indicating a significant improvement in the number of repetitions of trunk lifts), suggesting that the training had a positive impact on core strength.

#### 3.2.3. Back-Saver Sit and Reach Test

The hamstring muscle flexibility improved from an average of −7.86 to a −3.73 cm, showing a statistically significant gain in flexibility (*p* < 0.001). Cohen’s d = 1.25 (large effect, indicating a significant improvement in participants’ flexibility as measured by the sit and reach test), indicating increased mobility and flexibility after training.

#### 3.2.4. Shoulder Stretching Test

The shoulder muscle flexibility test showed an average improvement from −12.00 to −8.26 cm on the right arm and −12.11 to −7.76 cm on the left arm, showing statistically significant gains in flexibility for both the right (*p* < 0.05) and left shoulder (*p* < 0.01). Cohen’s d (R) = 0.64 and Cohen’s d (L) = 0.81, and both effect sizes are moderate to large, showing a significant improvement in shoulder flexibility after training.

#### 3.2.5. Aerobic Fitness Test—6MWT

The average gait speed increased from 1.10 to 1.35 m/s, showing a statistically significant increase in gait speed (*p* < 0.001). The use of the ankle foot orthosis was permitted during testing. Cohen’s d = 1.60, showing a very large effect size, indicating a significant improvement in performance on the 6 min walk test. This suggests an increase in physical capacity and endurance of the participants.

#### 3.2.6. Aerobic Endurance Test—Step Test

VO_2_ max was measured using the step test. The mean VO_2_ max value improved from 52.57 to 56.22 mL/kg/min, showing a statistically significant increase (*p* < 0.01). The use of the ankle foot orthosis was allowed during testing. Cohen’s d = 0.96, showing a large effect size and suggesting a considerable increase in participants’ aerobic capacity after training.

### 3.3. Depression, Anxiety, and Stress (DASS-21)

**Figure 1:**
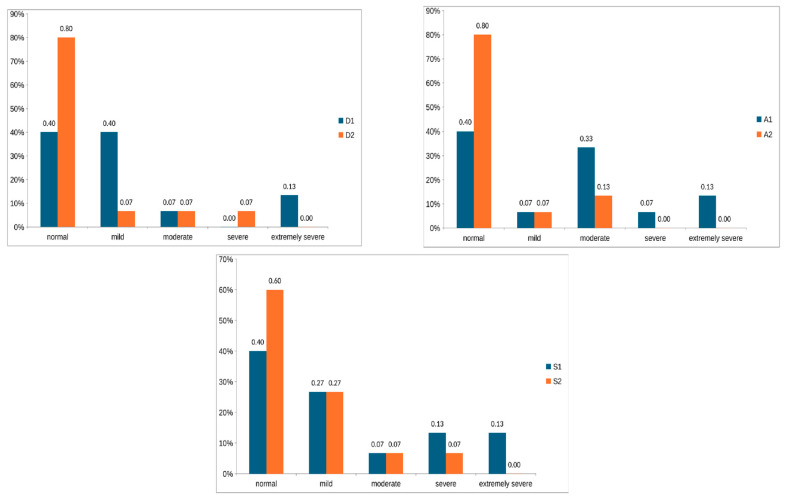
Participants’ values for depression, anxiety, and stress from DASS-21 scale. D1, A1, and S1 correspond to the values of the first assessment on the depression, anxiety, and stress scales, respectively. D2, A2, and S2 correspond to the values of the second assessment, after 3 months of intervention, on the depression, anxiety, and stress scales, respectively.

Figure 1 shows the values of the initial and final evaluations of the participants with CP on the DASS-21 scale for depression, anxiety, and stress.

Table 4 presents participant’s descriptive values for depression, anxiety, and stress subscales and the mean differences pre- and post-intervention with exercise, calculated with Pearson’s chi-square test.

#### 3.3.1. Depression

In the first assessment moment (D1) before intervention with exercise, two adolescents presented results suggestive of extremely severe depression, one moderate, six mild, and six within the normal range (*n* = 15). In the second assessment moment (D2), after intervention with exercise, one adolescent presented results suggestive of severe depression, one moderate, one mild, and twelve within the normal range (*n* = 15). A relevant improvement in perceived depression was observed due to exercise intervention; however, this result is statistically seen as marginal (*p* = 0.058).

#### 3.3.2. Anxiety

In the first assessment moment (A1), two adolescents presented results suggestive of extremely severe anxiety, one severe, five moderate, one mild, and six within the normal range (*n* = 15). In the second assessment moment (A2), two adolescents presented results suggestive of moderate anxiety, one mild, and twelve within normal limits (*n* = 15). A statistically significant improvement in anxiety was observed due to exercise intervention (*p* ˂ 0.05).

#### 3.3.3. Stress

In the first assessment moment (E1), two adolescents presented results suggestive of extremely severe stress, two severe, one moderate, four mild, and six within the normal range (*n* = 15). In the second assessment moment (E2), one adolescent presented results suggestive of severe stress, one moderate, four mild, and nine within normal limits (*n* = 15). A statistically significant improvement in stress management was observed due to exercise intervention (*p* ˂ 0.05).

### 3.4. Salivary Cortisol

Four samples were collected for each adolescent before and after the first day of physical activity (moments 1.1 and 1.2 here) and before and after the last day (moments 2.1 and 2.2). The average values obtained were moment 1.1 = 7.7 pg/mL; moment 1.2 = 5.7 pg/mL; moment 2.1 = 6.5 pg/mL; and moment 2.2 = 4.1 pg/mL. Although there was a reduction in the mean salivary cortisol values, when we compared moments 1.1 and 1.2 and moments 2.1 and 2.2, this reduction was not statistically significant (*p* = 0.222 and *p* = 0.091, respectively). Globally, no significant statistical differences were found for the salivary cortisol biological marker pre- (moment 1.1) and post-intervention (2.1) as a result of exercise (*p* = 0.668). Cohen’s d = −6.32, showing a large negative effect, suggesting a reduction in salivary cortisol levels after training. This may indicate a reduction in participants’ stress levels.

### 3.5. Quality of Life

The KIDSCREEN-10 questionnaire was answered by adolescents (self-assessment) and one of their parents or legal guardians. Our sample included eleven mothers, two fathers, and two sisters. Due to difficulty understanding the questions, two teenagers needed help from their family members to answer the questionnaire. The KIDSCREEN and KIDSCREEN proxy test values are provided in Table 3. A small reduction trend in the mean values was observed; however, such reduction was not statistically significant. Cohen’s d = −0.29, showing a small, negative effect, indicating that there was a slight reduction in quality-of-life scores. The change is small and may not be clinically relevant. Cohen’s d = 0.00 for the proxy test, as there were no changes in the quality-of-life scores reported by the proxies (observers), indicating that the observers’ perception of the participants’ quality of life remained unchanged.

## 4. Discussion

This study allowed us to investigate the effects of a mixed-method exercise program on physical fitness, stress, anxiety, and health-related quality of life in adolescents with CP. Muscle strength, aerobic, and muscle flexibility exercises were offered face-to-face and online in a live exercise program built up for adolescents with CP. No adverse events were reported during the months of intervention.

The most significant results were related to physical fitness tests, showing a direct cause-and-effect relationship with the exercise program in the mixed method. The mixedexercise program resulted in gains in strength, flexibility, and aerobic endurance, characterized by an increase in average walking speed and average VO_2_ max. These results highlight the importance of the exercise program’s specificity, which should be directed toward the predefined set objectives. Furthermore, it can confirm that the frequency and intensity of the training were adequate for the participants.

Baseline results from the DASS-21 scale identified the occurrence of negative emotional experiences of depression, stress, and anxiety in adolescents with CP, but also provided evidence for the positive influence of physical exercise and significant differences found when compared with post-intervention values. The present study also shows a reduction in mean salivary cortisol levels after physical exercise, although this is not statistically significant.

Regarding quality of life, KIDSCREEN-10 classifies values as within or outside the expected range for interpretation purposes. The test presents an international table of values, where the expected average result for the age group of 12 to 18 years would be 47.21 ± 8.98 for girls and 49.97 ± 9.40 for boys [39]. The average results found in the present study, both before and after the intervention, were below the average of the international table. In spite of the physical fitness and reported emotional state improvement, the quality-of-life perception did not change significantly. Quality of life refers to perceptions of one’s position in life, functioning, and broad assessment of well-being across multiple domains, where physical and emotional well-being are parts of a wider construct [39]. Possibly, physical and emotional changes achieved with the program were not sufficient to impact the final outcome on the assessment scale.

Physical exercise programs for adolescents with CP are diverse but predominantly face-to-face, and the systematic reviews that investigated the effects of muscle strengthening with adolescents with CP found gains in strength and gait parameters [16,42,43]. Other reviews investigating the effects of aerobic exercise also reported gains in gait parameters [19], with a positive impact on the cardiorespiratory fitness system and increased VO_2_ max [44]. All studies included in the systematic reviews were face-to-face and varied in the number of studies included, the number of participants, exercise protocols, and follow-up. Concerning negative emotional experiences assessed using the DASS-21 scale, the results found in this study corroborate previous studies on the identification of the occurrence of negative emotional experiences of depression, stress, and anxiety in adolescents with CP [4] and the positive influence of physical exercise with changes in those emotional experiences [45]. The present study did not show a significant reduction in average salivary cortisol levels after physical exercise. Studies conducted with adolescents without CP indicate that physical exercise does not show significant differences regarding the variation in salivary cortisol levels [46,47]. No studies were found with participants with CP to allow direct comparison and discussion. However, the present study’s trend is similar to that found in other studies with adolescents without disability. Additionally, we may also associate the occurrence of significant differences with the length of the exercise program and its relationship with the exercise intensity. Due to the psychomotor characteristics of CP, it is more difficult to reach higher levels of aerobic exercise, close to the anaerobic threshold, for an extended period, so the positive effects of aerobic exercise will probably take more time to become statistically significant, explaining why a 12-week-long program may not be long enough to maximize the potential effects of exercise in adolescents with CP. Additional studies are needed to answer some of the questions highlighted in the present study. Concerning quality of life, our result is similar to that found in the systematic review by Soares, Gusmão, and Souto (2023) [19], where face-to-face aerobic exercises also did not improve quality of life. The review verified the effectiveness of aerobic exercises, and the results show a significant effect on aerobic capacity, balance, gross motor function, mobility, and participation. However, aerobic exercise did not impact quality of life. In this review, the included studies used varied quality-of-life assessment scales, and the samples were also small. The authors report that definitive conclusions have yet to be reached as this could lead to underestimation of the heterogeneity and, consequently, low uncertainty of the available evidence [19]. On the other hand, the study by Maher, Toohey, and Ferguson (2015) found a positive association between physical activity, social and physical quality of life, and happiness in young people with cerebral palsy [48]. As mentioned previously, quality of life is a broad construct, referring to perceptions of one’s position in life [39]. Gains in health-related quality of life may be valued by some and not by others, depending on the position these aspects occupy in each individual’s life. A recent scoping review [49] highlighted that systematic reviews and meta-analyses on the effects of physical exercise in adolescents with CP were diverse regarding the exercises studied, the number and type of studies as well as the number of participants in each study. Therefore, we believe that further studies are still necessary. 

To our knowledge, no previous studies have evaluated the effects of exercise in adolescents with CP using a mixed-method approach. However, a previous study that evaluated the effectiveness of an online tool replacing a paper-based exercise program indicated that this method was similar to paper for adherence and outcome measures for children and adolescents with CP [24]. A study that compared a face-to-face and online exercise program to relieve neck pain in young adults found a positive result in reducing anxiety in both groups [50]. Another study that implemented an online physical activity program for 12 weeks with obese children showed gains in physical fitness and a reduction in BMI [51]; however, those previous studies did not perform a mixed online and in-person program, as was the case in the present study.

We recognize that it can be challenging for adolescents with CP to follow the exercise guidelines’ recommendations, as personal and environmental factors are identified barriers. Some adolescents report being embarrassed to exercise in public places, feeling excluded in gyms, a lack of opportunity, a lack of time, difficulty in transportation, financial problems, and difficulty finding an available companion [22,23]. The companion, who, in turn, needs to have free time, and the frequency of the activity can often be a source of family stress [22,23]. Our study identified financial difficulties and the lack of a companion as the main reasons for missing face-to-face appointments. The literature also describes such factors as barriers to the practice of physical activity in adolescents [23], which is one reason why we believe it is important to study new strategies to facilitate access to physical exercise for this population. On the other hand, although it was greatly encouraged during the COVID-19 pandemic, exclusively online exercise in adolescents with cerebral palsy has not been studied for its long-term effect. We were concerned that exclusively online methods could compromise interaction between adolescents.

This mixed-method exercise program proved to be effective in improving physical fitness. This is a particularly relevant outcome because many patients have a sedentary lifestyle, increasing risk factors for cardiovascular disease [15]. Additionally, this approach allowed activities that encouraged adolescent interaction, which, when combined with exercise, may have contributed to the changes observed in their emotional state. We believe that the mixed method is promising, alternating the use of the online tool and face-to-face interaction, with the advantages of reducing expenses and travel time to the gym and, most importantly, maintaining interaction among adolescents. The mixed method can also be an alternative to encourage adherence to physical activity. However, this study did not aim to characterize adherence to the method due to the difficulties inherent in the study design. Future studies with a randomized method and larger samples may be more appropriate to test such adherence.

This study has some limitations. Patients were allowed to maintain conventional physical therapy and complete any parallel physical activity in addition to the intervention, which may have impacted the results. However, there were no differences in outcomes between patients who performed additional physical activity and those who did not. Due to the study design (case series) and the corresponding limited sample size, it was not possible to compare the effect of this specific exercise therapy program with other type of interventions or a control group, limiting the generalizability of the results. A randomized controlled trial with long-term outcomes is needed to determine whether this specific program is better than other programs using a control group. Future directions for this type of intervention research include a follow-up to better understand the long-term sustainability of the improvements in physical fitness and emotional well-being variables.

## 5. Conclusions

The 12-week mixed exercise program (face-to-face and online) resulted in gains in physical fitness, muscular strength, muscular flexibility, and aerobic endurance, characterized by an increase in average walking speed and average VO_2_ max in adolescents with ambulatory CP. The participants also reported a positive change in their perception of emotional states of depression, anxiety, and stress on the DASS-21 scale. The present results may contribute to the scope of evidence-based physical exercise guidelines for adolescents with CP, providing healthcare professionals with a clinical tool to help increase physical activity levels in this population.

## Figures and Tables

**Table 1:** Overview of the physical exercise program.

Exercise Type/(Duration)	Workout	Description	Category
Warm-up (Three minutes)	March on the spot	The teen raises each foot alternately two inches off the ground. Teens may need support.	Face-to-face and online
Warm-up (One minute)	Shoulder rolls	Teens should shrug and raise both shoulders without bending their head or neck then rotate their shoulders in a circle, bringing their shoulder blades together and releasing them to propel themselves forward. This should be repeated in the other direction.	
Warm-up (One minute)	Torso rotation	The teenager should place their hands in front of them with their elbows bent and turn their upper body to the left and right, keeping their knees forward without twisting their legs. Their cadence should be adjusted according to their preference.	
Warm-up (Five minutes)	Stationary cycling	Stationary cycling.	Face-to-face
Aerobic Exercise (Five minutes)	Step box with target	The teenager should climb one step, perform plantar flexion of the ankle, and try to reach the target fixed to the wall above their head to score points. Teens may need support.	Face-to-face and Online
Aerobic Exercise (Five minutes)	Circuit with basketball	The teenager will walk as fast as possible, deviating from five cones and through five bars on the floor. At the end of the circuit, they will receive a ball and try to throw it into the basket, earning a point if they get it in the basket.	
Aerobic Exercise (Ten minutes)	“Burned”	This is a popular local game. To play, it is necessary to divide the group into two teams. The player with the ball must throw it, trying to hit (burn) a person on the other team. Whoever is burned leaves the game. The team that manages to burn the entire opposing team first wins.	Face-to-face
Strength Training	Elastic band workout	Leg extesion (seated), glute bridge, clamshell, plantar flexion, lateral band walk, seated abduction, standing bicep curl, overhead tricep extension, pull apart, lying pull over, forward raise, and lateral raise (2 sets of 10 repetitions, progressively increased by changing the elastic band).	Face-to-face and online
Strength Training	Strength training	Plank, low squat, wall sit, isometric push-up, bridge, and calf stretch against the wall (1 to 3 sets of 10 to 15 repetitions of each of the exercises (progressively increased)).	
Flexibility/Relaxation (Ten minutes)	Yoga class	Downward dog on a chair (Uttana Shishosana), child’s pose (Balasana), bound angle pose (Baddha Konasana), and cat–cow (Marjaryasana-Bitilasana).	Face-to-face and online
Flexibility/Relaxation (Ten minutes)	Stretching	Triceps stretch, arms and abs stretch, standing quad stretch, hamstring and calf stretch, and figure four stretch.	

**Table 2:** Main characteristics of the CP participants in the study.

Participant	1	2	3	4	5	6	7	8	9	10	11	12	13	14	15
CP Dist.	HR	D	QM	HL	D	HL	D	D	QM	D	D	D	D	QM	QS
GMFCS	I	I	III	I	II	I	I	I	II	I	III	I	I	II	II
AFO	N	N	N	N	N	N	N	N	N	N	N	N	N	Y/R	N
Age	13	15	16	12	15	12	14	16	15	17	15	13	12	13	12
Sex	M	F	F	F	F	F	M	M	M	M	F	M	M	F	M
Total Frequency %	91.6	95.8	83.3	83.3	100	87.5	75	87.5	100	91.6	91.6	75	75	100	100
Frequency Face-to-face/online %	91.6/91.6	91.6/100	83.3/83.3	75/91.6	100/100	83.3/91.6	75/75	83.3/91.6	100/100	83.3/100	91.6/91.6	75/75	66.6/83.3	100/100	100/100
PA	Y	N	N	N	N	N	N	N	N	Y	Y	N	N	N	Y
Recreation	Y	Y	N	Y	Y	Y	Y	Y	Y	Y	Y	N	Y	Y	N
Transport	Bus	Car	Car	Bus	Car	Car	Bus	Bus	Bus	Bus	Car	Car	Bus	Bus	Car

CP DIST.: distribution of cerebral palsy; HR: hemiplegia (right); D: diplegia; HL: hemiplegia (left); QM: quadriparetic (mixed); QS: quadriparetic (spastic); GMFCS: Gross Motor Function Classification System levels I to III; AFO: ankle foot orthosis—Y: yes, N: no; AGE: age; Sex—F: female, M: male; Frequency %: frequency of participation in the activity, in percentage; PA: physical activity—Y: yes, N: no; Recreation: included in recreational activity—Y: yes, N: no.

**Table 3:** Mean and standard deviation values, and mean differences pre- and post-intervention with exercise, of the body composition, physical fitness, salivary cortisol levels and quality of life, calculated with a *t*-test.

Outcome	Combined Training Group (*n* = 15)			Sig. (Two-Way)	
	Pre	Post	Δ_mean_ ± SD	*t*	*p* Value
Body mass (kg)	48.4 ± 12.82	48.4 ± 13.06	0.00 ± 0.86	0.045	0.965
Height (cm)	154.0 ± 0.11	154 ± 0.11	0.00 ± 0.00	−1.000	0.334
BMI (kg/m^2)^	20.1 ± 4.13	20.1 ± 4.15	0.00 ± 1.22	0.101	0.921
WC (cm)	71.1 ± 11.22	70.5 ± 10.76	−0.56 ± 1.91	1.145	0.271
Modified curl (rep)	22.8 ± 17.66	62.1 ± 12.52	39.40 ± 18.53	−8.235	0.000 *
Trunk lift (rep)	23.1 ± 7.52	28.5 ± 6.34	5.36 ± 5.03	−4.126	0.001 *
Seat and reach (cm)	−19.2 ± 9.77	−11.4 ± 9.10	7.86 ± 6.30	−4.836	0.000 *
Shoulder stretch (R)	−12.0 ± 11.34	−8.2 ± 10.73	3.78 ± 5.94	−2.432	0.029 **
Shoulder stretch (L)	−12.1 ± 10.44	−7.7 ± 7.82	4.34 ± 5.35	−2.928	0.013 **
Step test (VO_2_ max)	52.6 ± 11.93	56.2 ± 11.85	3.65 ± 3.82	−3.696	0.002 *
6MWT (m/s)	1.10 ± 0.32	1.35 ± 0.34	0.24 ± 0.15	−5.975	0.000 *
Salivary cortisol (µg/dL)	7.7 ± 5.53	6.5 ± 5.34	−1.2 ± 0.19	0.439	0.668
KIDSCREEN (score)	38.6 ± 6.17	37.4 ± 7.73	−1.50 ± 5.13	1.002	0.329
KIDSCREENPROXY	41.6 ± 5.96	41.6 ± 5.96	0.00 ± 0.00	0.569	0.579

** Significant for *p* ˂ 0.05, * Significant for *p* ˂ 0.01. BMI: body mass index; WC: waist circumference; VO_2_ max: maximum oxygen volume; 6MWT: six-minute walk test; kg: kilogram; cm: centimeters; kg/m^2^: kilogram per square meter; rep: repetition; R: right; L: left; m/s: meters per second; µg/dL: micrograms per milliliter.

**Table 4:** Dass-21 scale of depression, anxiety, and stress—Pearson proportion analysis test.

Outcome	Df	*n*	Pearson Value	Likelihood Ratio	*p* Value
Depression	9	15	16.458	13.424	0.058
Anxiety	8	15	17.000	13.827	0.030 **
Stress	12	15	23.229	20.557	0.026 **

** Significant for *p* < 0.05.

## Data Availability

The authors confirm that the data supporting the findings of this study are available within the article.
